# The association between the lack of safe drinking water and sanitation facilities with intestinal *Entamoeba spp* infection risk: A systematic review and meta-analysis

**DOI:** 10.1371/journal.pone.0237102

**Published:** 2020-11-04

**Authors:** Hamid Atabati, Hamid Kassiri, Ehsan Shamloo, Mitra Akbari, Ali Atamaleki, Fatemeh Sahlabadi, Nguyen Thi Thuy Linh, Ali Rostami, Yadolah Fakhri, Amin Mousavi Khaneghah

**Affiliations:** 1 Department of the environment faculty of fishery and environment, Gorgan University of agriculture and natural resources sciences, Golestan Province, Gorgan, Iran; 2 Department of Medical Entomology, School of Health, Ahvaz Jundishapur University of Medical Sciences, Ahvaz, Iran; 3 Noncommunicable Diseases Research Center, Department of Food Science and Technology, Neyshabur University of Medical Sciences, Neyshabur, Iran; 4 Amiralmomenin Hospital, School of Medicine, Guilan University of Medical Science, Rasht, Iran; 5 Department of Environmental Health Engineering, Student Research Committee, Shahid Beheshti University of Medical Sciences, Tehran, Iran; 6 Institute of Research and Development, Duy Tan University, Danang, Vietnam; 7 Faculty of Environmental and Chemical Engineering, Duy Tan University, Danang, Vietnam; 8 Infectious Diseases and Tropical Medicine Research Center, Health Research Institute, Babol University of Medical Sciences, Babol, Iran; 9 Social Determinants in Health Promotion Research Center, Hormozgan Health Institute, Hormozgan University of Medical Sciences, Bandar Abbas, Iran; 10 Department of Food Science, Faculty of Food Engineering, University of Campinas (UNICAMP), Campinas, São Paulo, Brazil; Taipei Medical University/Medicine, TAIWAN

## Abstract

Intestinal protozoa infections are responsible for considerable morbidity and mortality, especially where the exposed population suffers from a lack of drinking water and sanitation facilities. In this study, the association between the lack of safe drinking water and sanitation (toilet) facilities with intestinal *Entamoeba spp* infection in the children (5–11 years), adult (18–55 years), and all age (5–55 years) were assessed. For this purpose, some of the international databases such as Scopus, PubMed, Web of Science, and Embase were screened to up to 7 June 2019 in order to retrieve the related citations. Also, the pooled odds ratios (ORs) following 95% confidence intervals (CIs) were calculated using a random-effects model. Twenty-nine articles with 36 studies were included while the OR extracted or calculated by using 2 × 2 contingency tables. However, the ingestion of contaminated water insignificantly can increase the odds ratio (OR) of *Entamoeba spp* infection (OR 1.01, (95% confidence interval [CI] 0.58 to 1.43), no access to sanitation (toilet) facilities significantly can increase odds of *Entamoeba spp* infection (OR 1.18, 95% CI 1.05 to 1.32). The meta-regression analysis showed that over time, odds of intestinal *Entamoeba spp* infection increased in both lack of safe drinking water (Coefficient: 3.24, P-value < 0.01) and sanitation (toilet) facilities (Coefficient: 2.36, P-value < 0.05) subgroups. Considering the findings, lack of safe drinking water resulted in a further increase in intestinal *Entamoeba spp* infection among adult (OR: 2.76), children (OR = 0.57) and all age groups (OR: 1.50), and also lack of sanitation (toilet) facilities resulted in further increase intestinal *Entamoeba spp* infection in children (OR: 1.06), adult (OR: 1.26) and all age (OR: 1.16). In this context, the lack of safe drinking water and sanitation facilities (toilet) was associated with a high risk of intestinal *Entamoeba spp* infection. Further attempts to providing public health facilities can control the prevalence of intestinal *Entamoeba spp*.

## 1. Introduction

While the intestinal protozoa infections with some types of Entamoeba spp such as *Entamoeba histolytica*, *Giardia intestinalis*, *Strongyloides stercoralis*, and *Crypto sporidium spp*, they can be considered as one of the serious human health threats due to the notable malnutrition, mortality and morbidity rates in the worldwide [1–6]. In this context, according to the World Health Organization (WHO), the endemic of schistosomiasis occurred in 78 countries, which resulted in further treatments for about 261 million people [7]. The prevalence of *Giardia intestinalis* among the developing and developed countries was estimated as 20–30% and 2–3%, respectively, which can be accounted for serious health risks [4, 8]. While the high prevalence of *Giardia intestinalis* among developing was highlighted, the higher prevalence of *Cryptosporidium spp* in the people infected with HIV (which mainly are found in developing countries particularly concentrated in African continent) highlighted the dangerous consequences of Intestinal protozoa infections [9, 10]. Moreover, amoebiasis due to *Entamoeba spp* can cause ~ 100,000 deaths annually, hence amoebiasis is one of the main global parasitic infections [2, 3]. However, the information regarding the burden of the disease caused by the intestinal parasitic is rare, probably due to the difficulty of accurate diagnosis [11]. Furthermore, due to amoebiasis and cryptosporidiosis 2.2 million and 8.4 million disability-adjusted life years (DALYs) were reported as in 2010 [12]. Also, DALYs related to both intestinal protozoa and schistosomiasis infections were estimated at about 26.1 million persons in 2015 [13].

While the environmental fecal contamination, poor hygienic standards, and lack of sanitation facilities are associated with intestinal parasitic infections (IPIs) [14, 15]. Also, socio-economic and ecological factors have a considerable effect on the burden of IPIs [16, 17]. For instance, the calculated risk of IPIs for children who are living in under developing countries (low and middle income) was recognized as high, mainly due to lack in providing safe drinking water and sanitation facilities [18–20]. In this regard, it was assumed that the health of more than 600 and 270 million children in preschool and school-age, respectively, can be threatened due to transmission intestinal parasites [21, 22]. In another hand, the IPIs are strongly associated with malnutrition, which contributes to one-third of all deaths of children under five age [23].

Since intestinal parasites can be transmitted through the oral-fecal route and contaminated water, the prevalence of intestinal infections countries that are facing the lack s in safe drinking water and suitable sanitation facilities like low and middle-income countries and rural areas is relatively high [24, 25]. Based on the reports, about 2.5 million and 780 million people have no access to sanitation facilities and safe drinking water, respectively [26]. Additionally, considerable geographical heterogeneity in the safe drinking water supply and sanitation facilities was observed in developing countries [27].

Since several contradictory studies have been conducted on the risk of intestinal *Entamoeba spp* infection in the communities with the lack of safe drinking water and sanitation facilities [28–40], a systematic review and meta-analysis can help us to elucidate the association of contaminated drinking water consumption and live in poor sanitation with the risk of intestinal *Entamoeba spp* infection.

Therefore, the current systematic review and meta-analysis were performed to determine the risk of intestinal *Entamoeba spp* infection based on the ingestion of the contaminated drinking water as well as poor sanitation facilities based on the age population (children, adults, and all age), besides the evaluation of studies quality. Also, a meta-regression analysis was approached to determine the associated risk of intestinal *Entamoeba Spp* infection over time.

## 2. Materials and methods

### 2.1. Protocol and search strategy

A systematic review and meta-analysis were conducted according to the Meta-analysis of Observational Studies in Epidemiology (MOOSE) guidelines [41] and Preferred Reporting Items for Systematic reviews and Meta-Analyses (PRISMA) [42]. In this regard, the main international databases, including Scopus, Web of Science, PubMed, and Embase, were screened to retrieve the related citations between up to 7 June 2019. Keywords and MeSH terms of “water” OR “toilet facilities,” OR “sanitation,” AND “risk factor, AND “odds ratio” OR “relative risk” AND “Intestinal Protozoa,” OR “*Entamoeba histolytica*,” OR “*Entamoeba dispar*,” OR“ *Entamoeba moshkovskii*.” were used. It is noteworthy, in this study *Entamoeba histolytica*, *Entamoeba dispar* and *Entamoeba moshkovski* considered as *Entamoeba spp*.

The title, abstract and full texts of obtained articles were screened for eligibility. Also, according to performed systematic review studies [43–47], the references list of articles were screened to collect more citations.

### 2.2. Inclusion and exclusion criteria

An article was included when it met the proposed inclusion criteria [48–51], including a). Full-text of the article in the English language published; b) prevalence of *Entamoeba histolytica*, *Entamoeba dispar*, and *Entamoeba moshkovskii* in stool, c) cross-sectional (c-s) and case-control (c-c) studies; (d) reporting the lack of sanitation facilities (toilets); e) investigation regarding the lack of safe drinking water. In this regard, the ecological, animal studies, genetic, theses, case reports, books, and review articles, and original studies with other languages (except English) were excluded. Studies that reported the risk factors after an outbreak and epidemic also were excluded. As chlorination cannot be considered as an efficient way to reduce and control of intestinal protozoa, studies that have described chlorination as water treatment have been excluded [52, 53].

### 2.3. Data extraction and quality assessment

The extracted data can be summarized as the study design, lacks safe drinking water and sanitation facilities, odds ratio (OR) following confidence interval (CI), country, study year, age group. Also, while the OR was presented among results of a study, same OR was included in the current, however, in the case of no reported OR, or presentation of results as text format and/or 2 × 2 contingency tables, further calculations by the following equation were performed to obtain the OR [54]:

$$OR\;=\;\frac{a\;\times d}{b\;\times c}$$ (1)

Where, a is the number of infected cases in the bad group; b, number uninfected cases in the bad group; c, number infected cases in good group and d, number uninfected cases in the good group [54].

Moreover, if a study contains both forms of OR (unadjusted and adjusted), the former one was employed to have an overview regarding the comparison between the cross-studies [4].

The quality of the studies was assessed according to the development and evaluation method [4, 55], Grading of Recommendations Assessment [56, 57], and diagnostic method to identify protozoa, assess the sanitation facilities and safe drinking water and finally strengths and limitations of the study. Studies with a total score of 4–6, 2–3, and lower 0–2 points were considered to be of high, moderate, and low qualities, respectively, while no studies were excluded according to performed quality assessment [56, 57].

### 2.4. Meta-analysis of data

A meta-analysis of ORs was conducted by STATA 14.0 (2015; STATA 14.0 Statistical Software, College Station, TX, USA). P-value < 0.05 was selected as statistical significant. Heterogeneity of studies was detected using Cochrane’s Q-test and *I*^*2*^ index as when *I*^*2*^ > 50%, heterogeneity is significant [58, 59]. When *I*^*2*^ index < 50% fixed effect model (FEM) but if *I*^*2*^ index > 50% random effect model (REM) was used [58]. In the current study, OR > 1 indicates an increasing effect of the lack of safe drinking water and sanitation facilities on the odds of infection with intestinal parasites. The Egger test was used to detect publication bias [58, 60]. A meta-analysis of data was performed based on lack of safe drinking water and sanitation facilities, age groups of the exposed population including children (5–11 years), adult (18–55 years), and all age (5–55 years), and quality of study subgroups. Also, meta-regression between ORs of safe drinking water lack and sanitation facilities with a year of publishing was performed in the non-linear model [50, 61].

## 3. Results

### 3.1. Study characteristics

**Fig 1:**
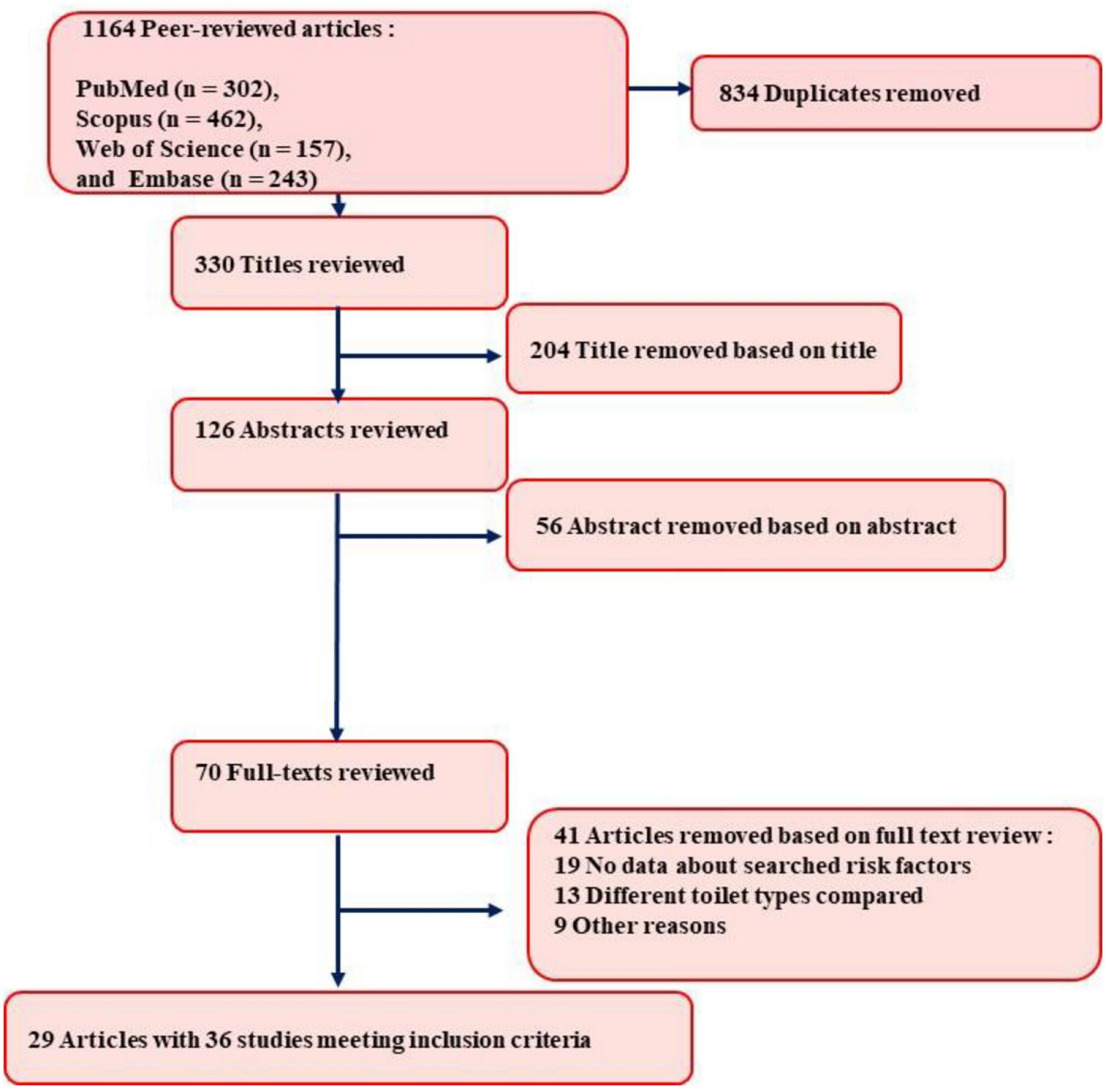
Schematic protocol for the selection and extraction of relevant studies.

In the initial step, 834 citations out of 1,164 collected articles were excluded due to duplication by the aid of EndNote X7^®^ software (Thomson Reuters, Toronto, Canada). Based on the assessment of the abstracts and titles, 456 articles were identified as suitable and were screened separately. Two hundred and sixty articles were excluded due to irrelevant content of title (n = 204) and abstract (n = 56). Full text of 70 articles was downloaded and considered, while 39 articles were removed due to no data about searched risk factors, different toilet types, and other reasons. Finally, 29 articles with 36 studies were included in the current study (Fig 1).

The rank order of countries based on number of published studies were Brazil (7) > India (6) > Ethiopia (3) > Cote dlvoire (2) ~ Kenya (2) ~ Lesotho (2) ~ Mexico (2) > Vietnam (2) ~ Colombia(1) ~ Ecuador (1) ~ Cuba (1) ~ Chile(1) ~ Cambodia(1) ~ Iraq (1) ~ Nigeria (1)~ South Africa (1) ~ Uganda (1) ~ Yemen(1). Overall, 36 studies, 25 studies were related to the risk of *Entamoeba spp* infection with lack of sanitation facilities, and 11 studies were associated with the issues raised by consuming contaminated drinking water. Also, among all studies, 34 and 2 studies were cross-sectional and case-control studies, respectively (S1 Table).

### 3.2. Meta-analysis of findings

**Fig 2:**
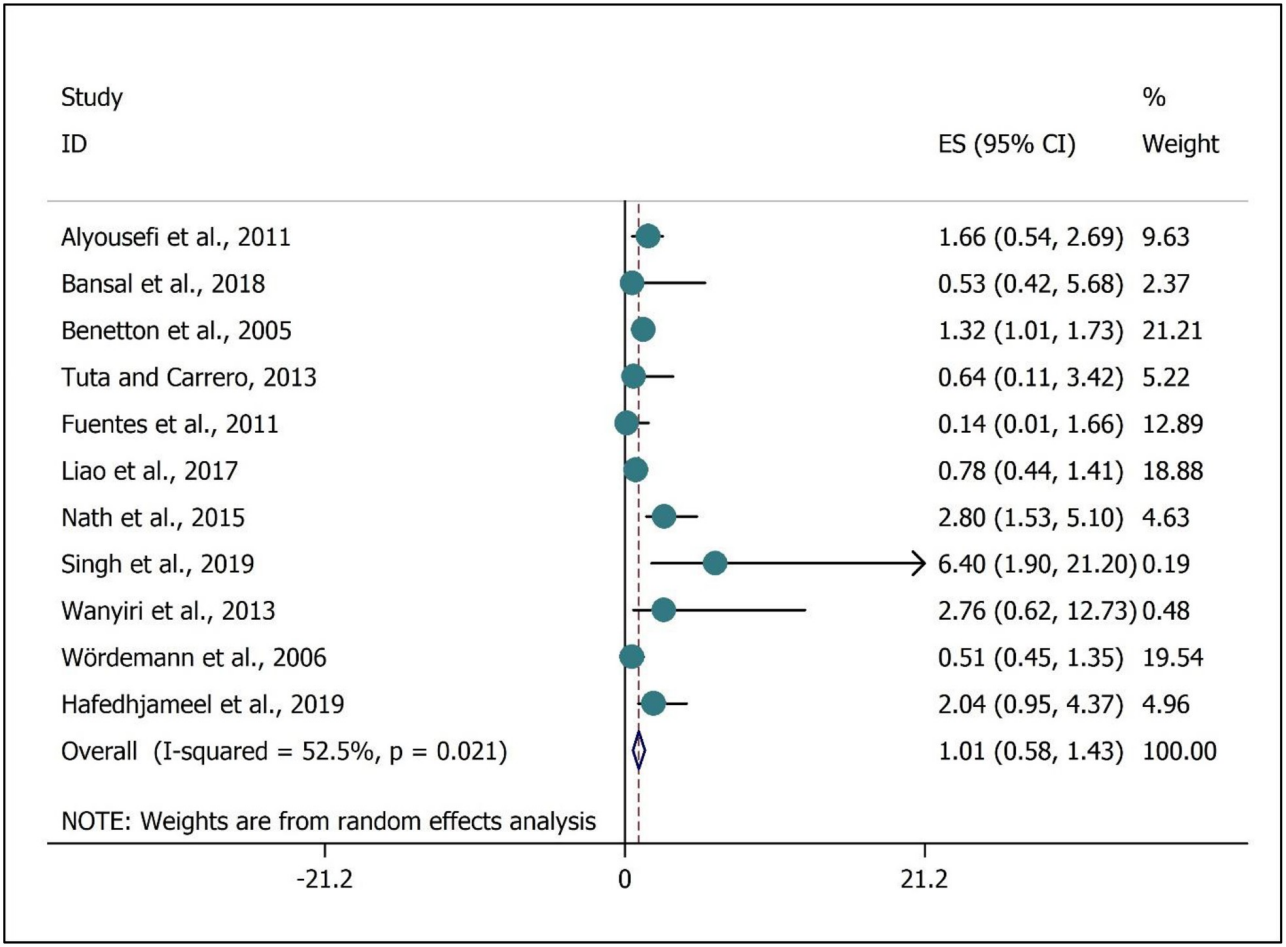
Meta-analysis of the association intestinal *Entamoeba spp* infection with lack safe drinking water. ES is Effect size.

**Fig 3:**
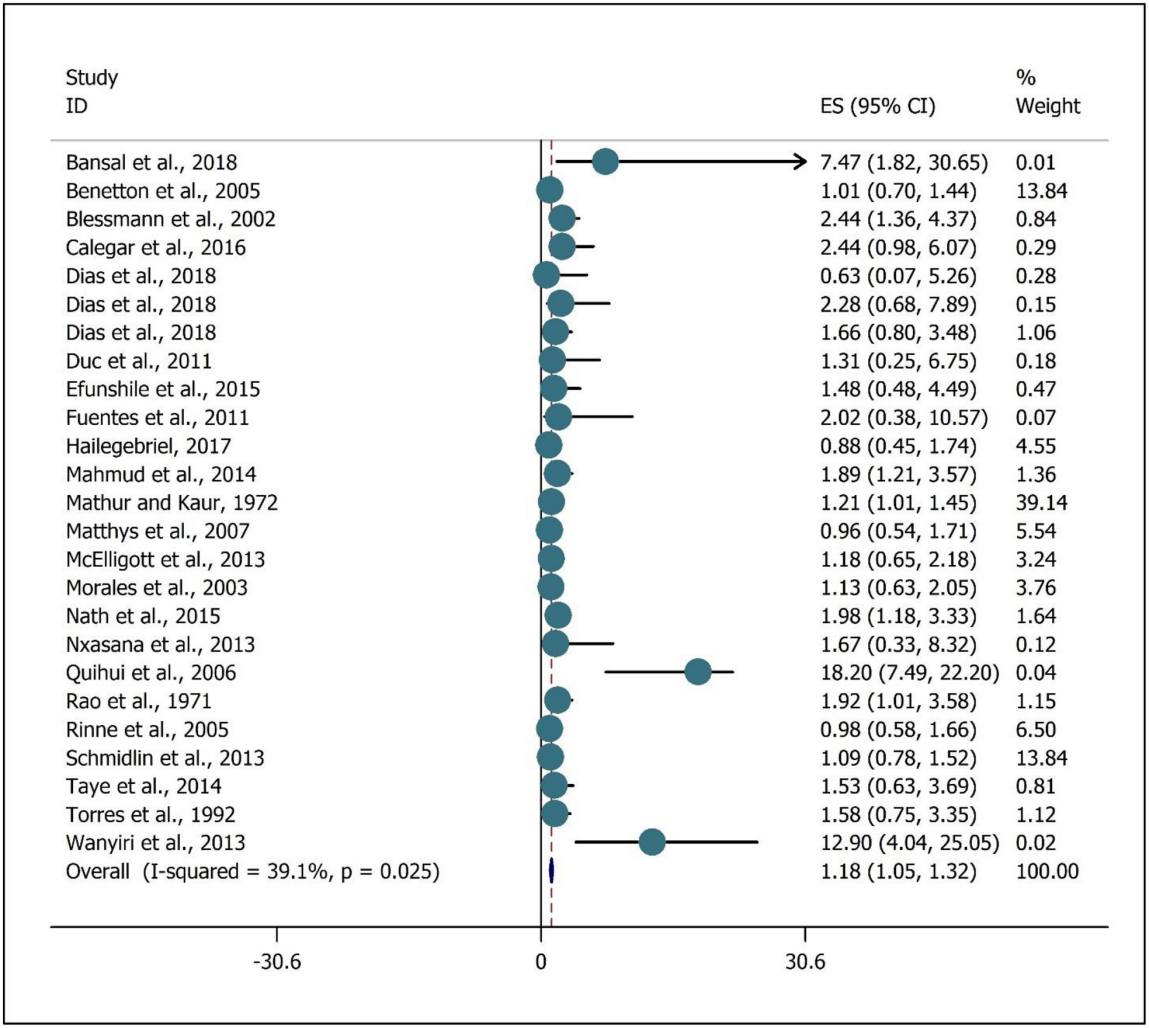
Meta-analysis of the association intestinal *Entamoeba spp* infection with lack sanitation facilities (toilet). ES is Effect size.

The meta-analysis result regarding the association between the intestinal *Entamoeba spp* infections and safe drinking water lack was presented in Fig 2. While a marginally insignificant positive correlation with high heterogeneity (*I*^*2*^ = 52.50%, P = 0.021) was noted (OR, 1.01; 95%CI 0.58 to 1.43) (Fig 2). The meta-analysis of the association between intestinal *Entamoeba spp* infections with a lack of sanitation facilities (toilet) was presented in Fig 3. While a significant positive correlation with low heterogeneity (I^2^ = 39.1%, P = 0.025) was determined (OR, 1.18; 95%CI 1.05 to 1.32) (Fig 3).

However, a negative insignificant association between the intestinal *Entamoeba spp* infection and lack safe drinking water in low-quality studies was observed (OR, 0.68; 95%CI 0.25 to 1.11), in moderate quality studies, a positive insignificant was observed (OR, 1.31; 95%CI 0.68 to 1.99) (S1 Fig). A positive insignificant association between the intestinal *Entamoeba spp* infection and lack sanitation facilities (toilet) in low-quality studies was noted (OR, 1.15; 95%CI 0.74 to 1.56). While in moderate quality studies, a positive significant association between the intestinal *Entamoeba spp* infection and lack sanitation facilities (toilet) was observed (OR, 1.19; 95%CI 1.04 to 1.36) (S2 Fig).

**Fig 4:**
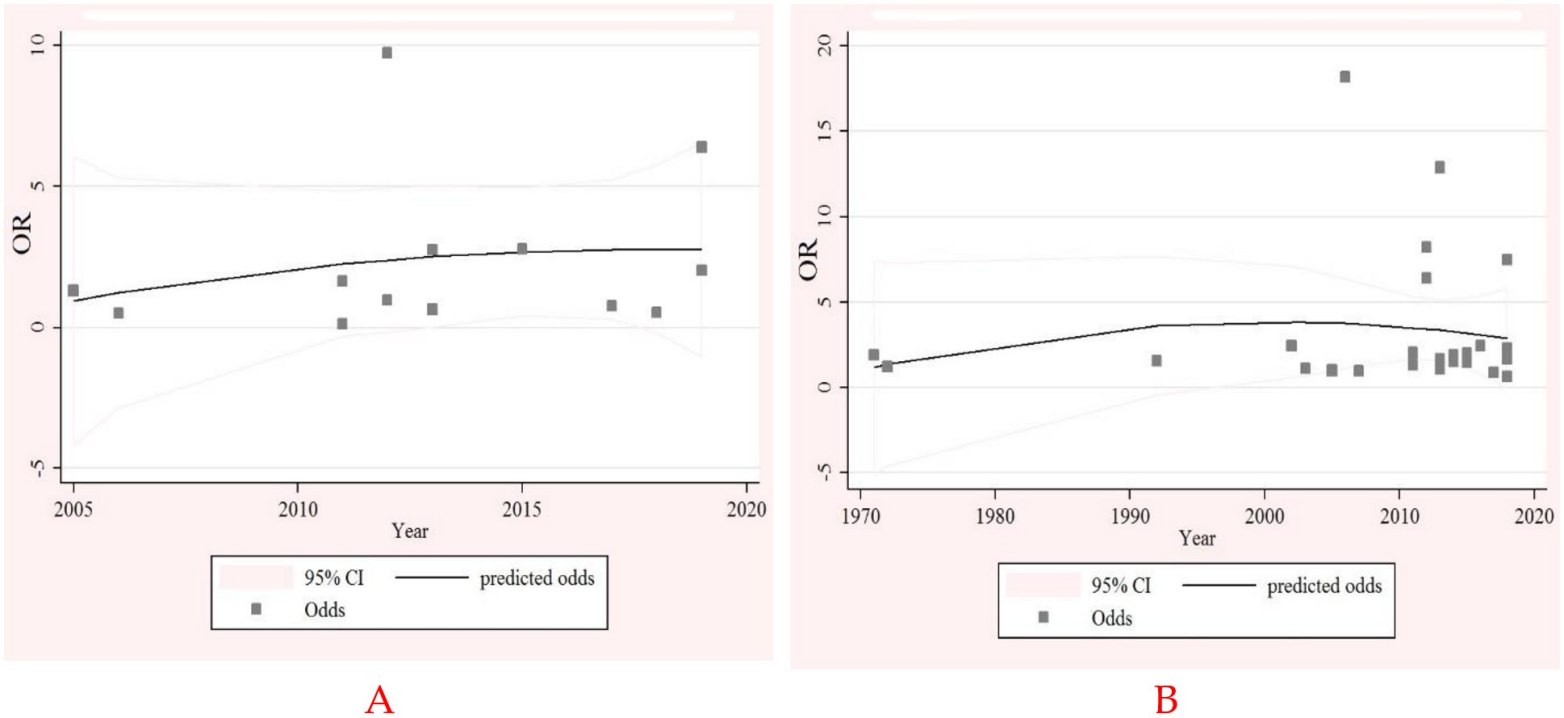
Meta-regression regarding the association of intestinal *Entamoeba spp* infection with year of publication study in the lack of safe drinking water (A) and lack sanitation (toilet) facilities (B) subgroups.

Although a negative significant association between the intestinal *Entamoeba spp* infection and the lack safe drinking water in children (OR, 0.57; 95%CI 0.27 to 0.87) was observed, a positive insignificant association among adult and all-age population (OR, 2.76; 95%CI 0.62 to 12.73), (OR, 1.50; 95%CI 1.08 to 1.93), respectively, was observed (S3 Fig). Additionally, a positive insignificant association between the intestinal *Entamoeba spp* infection and lack sanitation facilities (toilet) in children (OR, 1.06; 95%CI 0.71 to 1.40), positive significant in adult (OR, 1.26; 95%CI 1.05 to 1.48), and positive insignificant all age (OR, 1.16; 95%CI 0.95 to 1.37) was observed (S4 Fig). According to meta-regression analysis, a significant positive regression between *Entamoeba* spp infection and year of study in the safe drinking water lack subgroup was observed (Coefficient: 3.24, P-value < 0.01) (Fig 4A) and also regarding the lack sanitation (toilet) facilities subgroup (Coefficient: 2.36, P-value < 0.05) same observation was noted (Fig 4B). No significant publication bias regarding the lack of safe drinking water (P-value = 0.91) and lack of sanitation (toilet) facilities (P-value = 0.08) subgroups was reported based on the findings of publication bias test.

## 4. Discussion

In recent decades, the *Entamoeba spp*, due to the presence of invasive parasites within their species, particularly *Entamoeba histolytica*, has been investigated by several researchers [62]. In this context, the Amoebiasis as a threatening disease caused by mainly *Entamoeba histolytica* attracted notable attention [63]. However, in addition to *Entamoeba histolytica*, in many studies, other species like *Entamoeba dispar* have an effective role in the prevalence of intestinal diseases in humans as well as other animals [37, 64, 65]. In this context in most of the countries [63, 65, 66], except some countries around the Pacific Rim, the higher frequency of *Entamoeba dispar* rather than *Entamoeba histolytica* was noted. Moreover, a recent report published by Costa et al. (2018), in the city of Belo Horizonte located in southeastern Brazil, by applying the polymerase chain reaction, pointed out that the prevalence of *Entamoeba histolytica* is less than *Entamoeba dispar* [67].

The identified Entamoeba spp by using microscopic tools, are including *Entamoeba* histolytica and *Entamoeba* dispar, (pathogenic and non-pathogenic respectively) [68, 69]. However, other Entamoeba spp like *Entamoeba moshkovski and Entamoeba bangladeshi* were identified by some molecular methods [70]. Among the different *Entamoeba spp*, the distinction of *Entamoeba histolytica*, *Entamoeba dispar*, *and Entamoeba moshkovskii* by former method (microscope due to similar morphological) is difficult [67, 71]. Therefore, in the current systematic review, the association between the access to safe water and sanitation practices and infection of *Entamoeba* was assessed, without considering their species.

The ingestion of food/water contaminated with the cyst or through direct fecal-oral transmission can be considered as the main transmission route of the genus *Entamoeba* into the humans’ and animals’ bodies [72]. Indeed, *Entamoeba spp*. can distribute through the fecal-oral route with contaminated food and water [73]. In the current investigation, a marginally insignificant positive association between the intestinal *Entamoeba spp* infection and safe drinking water lack was observed (Fig 2). However, according to a previously published article, no association between the microbial water quality and infection of intestinal parasites was noted while this contrary results were attributed to the intervention of various factors [66]. As noted earlier, in addition to *E*.*hstolitica*, *Entamoeba dispar*, and *Entamoeba moshkovskii* can also play a remarkable role in the prevalence of intestinal parasites infection. Moreover, several factors are intervening on the transmission of protozoa into the body through contaminated water and food, which could influence the observed association between the contaminated drinking water and *Entamoeba* spp infection.

Maintaining water resources from microbial contaminants plays an important role in the control of transmitting diseases. In this regard, the parasitological quality of water collected from five different sources in rural areas of Tunisia was investigated, and the high level of intestinal parasite prevalence of (about 97%) mainly was correlated with protozoan [74]. Based on their findings, cleaning in the underground reservoirs and surface of rainwater collectors, changing the cover of reservoirs, and preventing run-off entrance to the reservoirs were proposed. In another study [75] in rural and urban areas of Jiroft, Iran, the quality of water and personal hygiene were recognized as the most important factors on the prevalence of parasites. Also, with applying of humans and animal feces as fertilizer or during food preparation with polluted hands and even by polluted water, this issue can be stimulated [76, 77]. Washing of hands before eating and after toilet, nail trimming [17, 78], consumption of well cleaned raw vegetables [37], and safe disposal of feces (toilet using) are the main sanitation practices that could reduce direct fecal-oral transmission. In most communities, the probable transmission of parasitic infections was increased by the lack of sanitation facilities, including not access toilets [79]. Sungkar et al. [80] conducted a cross-sectional study in Kalena Rongo village, Indonesia, where access to safe water and sanitary toilets was rare. This condition resulted in a lack of handwashing and taking a shower just once per week. After investigation, they found that about half of the residents were infected by protozoan diseases.

According to the findings of Nath et al. (2015a) who investigated the *Entamoeba spp*. in stool samples [81], the unhygienic toilet had a significant role in protozoan distribution. Moreover, discharged feces to open drains have a higher risk for infection [79]. As mentioned earlier, there are several factors that could affect sanitation, as classified into three levels of individual/family, social, and environmental. However, they have an inter-association within and between the levels.

### 4.1. Individual/family level

Based on the extracted data from literature, income [82], animal contact [83], family size [37], nutritional status [24, 84], age [85], gender [86], occupation statue [81], and some other cases, such as sexual act [87], genetic and immunity system of host [67, 74] were considered as the most effective factors.

Among the above-mentioned factors, poverty is usually caused by unemployment, and families by low-income are deprived of many primary life health standards such as insurance, education, safe water, and food, etc. [88, 89]. Most marginalized populations have low incomes, and because of low health services, zoonotic diseases are prevalent among their societies [90]. Due to the scavenging activities and the consumption of water and food with unknown sources, animals easily infected with parasitic infections. Therefore, direct contact with infected animals can increase the risk of animal-to-human transmission [30]. According to Liao et al. [91], based on 308 questionnaires collected from primary schools in Battambang, Cambodia, 95.1% of infected children kept animals at their homes. Also, Hemmati et al. [83] stated that direct contact with animals besides the drinking water quality is among the most important factors on the intestinal infection. However, in a report conducted in Kisumu, Kenya, only direct contact with a domestic animal was correlated by the distribution and diversity of intestinal parasites [82].

Additionally, the number of family members have a primary role in intestinal parasitic infection. In this regard, 5 times more risk was reported for the families with a population of more than 5 members, due to increase in people’s activity among the family, the risk can be increased [37].

Diet is an important factor against invasive pathogens. Hence, the deficiency in vital body elements leads to malnutrition, which results in the susceptibility of the body against various diseases [92]. Raja and Karthick [93] demonstrated that due to nutritional deficiency, alcoholics are more susceptible *to Entamoeba histolytica* infection. However, since iron is a vital element for *Entamoeba histolytica*, iron deficiency in women who have regular menstrual blood loss could prevent the risk of *Entamoeba histolytica* infection [94].

In most of the studies, a factor of age usually was related to the occupation type. Indeed, with an increase in age, especially since childhood, human activity, and communication due to attending school and work have increased [17]. In another investigation, age, unlike sex, statistically had a significant association with the prevalence of *Entamoeba histolytica* and *Entamoeba dispar*. In a study published in southern Brazil, a higher risk of intestinal protozoa (60%) was reported for the male while compared with a female which attributed to their occupation [95]. Based on the findings of Al-Mekhlafi et al. [17], the risk of infection in males was about 1.5 times higher than females.

### 4.2. Social level

Some factors such as economic development [17], culture [77], water/wastewater infrastructures [96], waste disposal [82], education [97], and governor policies [98] were considered. In this regard, among developed countries due to the availability of appropriate water treatment technologies and educated citizens a better condition for control and decrease of pathogenic infection can be expected. Although, occasionally, there are issues with the entrance of other country infected citizens [98]. Also, the economic development resulted in further improvements in the sanitation conditions such as infrastructures, education, incomes, water and food quality, vector control, and sewage and waste disposal [84, 99]. In this regard, due to the strong association between the high level of education and modern culture, better sanitation behaviors are expected [100, 101]. Proper education about sanitary and routes of infection can have a remarkable controlling role in disease prevalence [102]. According to Ben Ayed et al. (2018), the education of hygiene behaviors such as regular handwashing, safe storage, and consumption of water is crucial for keeping safe water resources in rural areas [99]. However, the larger communities need to have better management for water supply and sanitary disposal of wastes and wastewater [45, 74]. These goals require appropriate infrastructure, which is largely related to government policy and economics [63, 85, 98, 99].

### 4.3. Environmental level

Desertification, deforestation, and modification in regions besides long and short term climate changes are among the environmental variables that affect the distribution of parasites [76, 101]. Prolonged drought besides hot weather leads to the consumption of water from inappropriate sources which results in higher prevalence risk [103]. Accordingly, the summer had the highest, and winter had the lowest positive effect on the prevalence of protozoan infections. While according to another investigation, the prevalence of *Entamoeba dispar* and *Entamoeba histolytica* is increased in rainy seasons. Early precipitation through roof cleaning and submergence of drains can lead to water supply pollution [66]. Nath et al. after comparing the *Entamoeba histolytica* prevalence in pre-monsoon, monsoon, and post-monsoon seasons, concluded that monsoon season has the greatest role in the *Entamoeba histolytica* prevalence [65]. As argued above, in recent decades, climate change has become a concerning phenomenon. In some regions of the world, the phenomenon causes prolonged drought. On the other regions, heavy rain, and subsequently, a flood can result in a higher prevalence of these issues [104]. Although cold and heat stress due to their influences on the endocrine and immune systems, can change the survival status of parasites in the body of hosts [105].

Concerning the mentioned factors, it can be concluded that the prevalence of *Entamoeba spp*. is varied from country to country. However, based on the findings of the current investigation, the intestinal *Entamoeba spp* infection due to safe drinking water lack in Malaysia, Kenia and Iraq was higher than other countries and also due to lack of sanitation (toilet) facilities in Malaysia, Kenia and Mexico were higher than other countries. Food and water-related diseases, particularly, protozoan infections known as the main health problems in Peninsular Malaysia [106]. After investigating the intestinal parasitism among two indigenous groups in Malaysia, given that many indigenous peoples had farming activities, Chin et al. attributed the infection in these two indigenous peoples to the use of "night soil" as fertilizer [89]. Also, poverty and lack of appropriate water network systems were considered as intervention factors. In the case of Kenya which has not yet reached the Millennium Development Goals specified by the United Nations, using of safe drinking water in an urban and rural area of Kenya, were 82% and 57%, respectively [107]. Iraq, one of the Middle East and North African countries (MENA), considered as an arid or semi-arid region. Concerning the reports, this country had improved sanitation of 98% for urban areas and 82% for rural areas. While wastewater management infrastructures in Iraq were weak, and most wastewater treatment systems did not have the proper efficiency resulting in untreated wastewater discharged to the environment. In another word, about 70% of wastewater is discharged to the rivers without any treatment which can be resulted in a low microbial quality of water resources [108–110]. Parasitic infections are among the top 20 diseases in Mexico, and about 53% of peoples are affected by these problems. In a study carried out in states of Sonora and Oaxaca in Mexico, the results indicated that parasitic infection had a prevalence of 47.2% among children. Lack of education, poverty, highly populated families, direct animal contact, and malnutrition can be listed among the most important causes [111, 112]. At a glance, it can be said that all of the mentioned countries are among the developing countries.

## Conclusion

In this work for the first time, the risk of *Entamoeba spp* infection due to encounter public health parameters consist of lacks safe drinking water and sanitation facilities based on defined subgroups were meta-analyzed. The risk of *Entamoeba spp* infection due to the lack of safe drinking water increased, insignificantly; however, the risk of *Entamoeba spp* infection due to the lack of sanitation facilities increased significantly. The risk *Entamoeba spp* infection with the lack of sanitation facilities in the children’s age groups was insignificantly decreased, therefore, in order to obtain more accurate results, it needs to be conducted more studies on the children’s age group in the future. Overall, the current study showed that public health could have the main role in increasing the risk of *Entamoeba spp* infection. Therefore, it is recommended to be performed plans to increase public health in communities.

## Supporting information

S1 Checklist PRISMA 2009 checklist. (DOCX)

S1 Table Main characteristic of the included studies. (DOCX)

S1 Text (DOCX)

S1 Fig Meta-analysis of the association intestinal *Entamoeba spp* infection with lack safe drinking water based on quality of studies. ES is Effect size.
(DOCX)

S2 Fig Meta-analysis of the association intestinal *Entamoeba spp* infection with lack sanitation facilities (toilet) based on quality of studies. ES is Effect size.
(DOCX)

S3 Fig Meta-analysis of the association intestinal *Entamoeba spp* infection with lack safe drinking water based on age groups subgroup. ES is Effect size.
(DOCX)

S4 Fig Meta-analysis of the association intestinal *Entamoeba spp* infection with lack sanitation facilities (toilet) based on age group subgroup. ES is Effect size.
(DOCX)

## References

[1] de Glanville W, Thomas L, Cook EA, Bronsvoort BdC, Wamae N, Kariuki S, et al Household socio-economic position and individual infectious disease risk in rural Kenya. Sci Rep. 2019;9(1):2972 10.1038/s41598-019-39375-z 30814567 PMC6393457

[2] Lozano R, Naghavi M, Foreman K, Lim S, Shibuya K, Aboyans V, et al Global and regional mortality from 235 causes of death for 20 age groups in 1990 and 2010: a systematic analysis for the Global Burden of Disease Study 2010. The lancet. 2012;380(9859):2095–128. 10.1016/S0140-6736(12)61728-0 23245604 PMC10790329

[3] Feng Y, Xiao L. Zoonotic potential and molecular epidemiology of Giardia species and giardiasis. Clin Microbiol Rev. 2011;24(1):110–40. 10.1128/CMR.00033-10 21233509 PMC3021202

[4] Speich B, Croll D, Fürst T, Utzinger J, Keiser J. Effect of sanitation and water treatment on intestinal protozoa infection: a systematic review and meta-analysis. The Lancet Infectious Diseases. 2016;16(1):87–99. 10.1016/S1473-3099(15)00349-7 26404667

[5] Olsen A, van Lieshout L, Marti H, Polderman T, Polman K, Steinmann P, et al Strongyloidiasis–the most neglected of the neglected tropical diseases? Trans R Soc Trop Med Hyg. 2009;103(10):967–72. 10.1016/j.trstmh.2009.02.013 19328508

[6] Farrell SH, Coffeng LE, Truscott JE, Werkman M, Toor J, de Vlas SJ, et al Investigating the effectiveness of current and modified World Health Organization guidelines for the control of soil-transmitted helminth infections. Clin Infect Dis. 2018;66(suppl_4):S253–S9. 10.1093/cid/ciy002 29860285 PMC5982801

[7] WHO. World Health Organization, 2015. Schistosomiasis: number of people treated worldwide in 2013. Epidemiol Rec. 2015;90:25–32. 25638822

[8] Escobedo AA, Cimerman S. Giardiasis: a pharmacotherapy review. Expert Opin Pharmacother. 2007;8(12):1885–902. 10.1517/14656566.8.12.1885 17696791

[9] Ungar BL. Cryptosporidiosis in humans (Homo sapiens) Cryptosporidiosis of man and animals: CRC Press; 2018 p. 59–82.

[10] Jex AR, Smith HV, Nolan MJ, Campbell BE, Young ND, Cantacessi C, et al. Cryptic Parasite Revealed: Improved Prospects for Treatment and Control of Human Cryptosporidiosis Through Advanced Technologies⋆. Adv Parasitol. 77: Elsevier; 2011. p. 141–73. 10.1016/B978-0-12-391429-3.00007-1 22137584

[11] Ouattara M, N’Guéssan NA, Yapi A, N’Goran EK. Prevalence and spatial distribution of Entamoeba histolytica/dispar and Giardia lamblia among schoolchildren in Agboville area (Côte d’Ivoire). PLoS Negl Trop Dis. 2010;4(1):e574 10.1371/journal.pntd.0000574 20087416 PMC2800181

[12] Murray CJ, Vos T, Lozano R, Naghavi M, Flaxman AD, Michaud C, et al Disability-adjusted life years (DALYs) for 291 diseases and injuries in 21 regions, 1990–2010: a systematic analysis for the Global Burden of Disease Study 2010. The lancet. 2012;380(9859):2197–223. 10.1016/S0140-6736(12)61689-4 23245608

[13] GBD. DALYs and HALE Collaborators, 2015. Global, regional, and national disability-adjusted life-years (DALYs) for 315 diseases and injuries and healthy life expectancy (HALE), 1990–2015: a systematic analysis for the global burden of disease study 2015. Lancet. 2016;388:1603–58. 10.1016/S0140-6736(16)31460-X 27733283 PMC5388857

[14] Forson AO, Arthur I, Ayeh-Kumi PF. The role of family size, employment and education of parents in the prevalence of intestinal parasitic infections in school children in Accra. PLoS One. 2018;13(2):e0192303 10.1371/journal.pone.0192303 29415040 PMC5802905

[15] Shrestha J, Bhattachan B, Rai G, Park EY, Rai SK. Intestinal parasitic infections among public and private schoolchildren of Kathmandu, Nepal: prevalence and associated risk factors. BMC Res Notes. 2019;12(1):192 10.1186/s13104-019-4225-0 30925938 PMC6441203

[16] Oboth P, Gavamukulya Y, Barugahare BJ. Prevalence and clinical outcomes of Plasmodium falciparum and intestinal parasitic infections among children in Kiryandongo refugee camp, mid-Western Uganda: a cross sectional study. BMC Infect Dis. 2019;19(1):295 10.1186/s12879-019-3939-x 30935405 PMC6444856

[17] Al-Mekhlafi AM, Abdul-Ghani R, Al-Eryani SM, Saif-Ali R, Mahdy MA. School-based prevalence of intestinal parasitic infections and associated risk factors in rural communities of Sana’a, Yemen. Acta Trop. 2016;163:135–41. 10.1016/j.actatropica.2016.08.009 27515811

[18] Hellard ME, Sinclair MI, Hogg GG, Fairley CK. Prevalence of enteric pathogens among community based asymptomatic individuals. J Gastroenterol Hepatol. 2000;15(3):290–3. 10.1046/j.1440-1746.2000.02089.x 10764030

[19] Practices ACoI. Centers for Disease Control and Prevention CDC. Immunization of health-care personnel: recommendations of the Advisory Committee on Immunization Practices (ACIP) MMWR Recomm Rep. 2011;60:1–45. 22108587

[20] Harhay MO, Horton J, Olliaro PL. Epidemiology and control of human gastrointestinal parasites in children. Expert Rev Anti Infect Ther. 2010;8(2):219–34. 10.1586/eri.09.119 20109051 PMC2851163

[21] Hotez PJ, Kamath A. Neglected tropical diseases in sub-Saharan Africa: review of their prevalence, distribution, and disease burden. PLoS Negl Trop Dis. 2009;3(8):e412 10.1371/journal.pntd.0000412 19707588 PMC2727001

[22] Sakari SSW, Mbugua AK, Mkoji GM. Prevalence of Soil-Transmitted Helminthiases and Schistosomiasis in Preschool Age Children in Mwea Division, Kirinyaga South District, Kirinyaga County, and Their Potential Effect on Physical Growth. J Trop Med. 2017;2017. 10.1155/2017/1013802 PMC5613645 29138640

[23] Kyu HH, Stein CE, Pinto CB, Rakovac I, Weber MW, Purnat TD, et al Causes of death among children aged 5–14 years in the WHO European Region: a systematic analysis for the Global Burden of Disease Study 2016. The lancet child & adolescent health. 2018;2(5):321–37. 10.1016/S2352-4642(18)30095-6 29732397 PMC5928398

[24] Lin A, Ercumen A, Benjamin-Chung J, Arnold BF, Das S, Haque R, et al Effects of water, sanitation, handwashing, and nutritional interventions on child enteric protozoan infections in rural Bangladesh: A cluster-randomized controlled trial. Clin Infect Dis. 2018;67(10):1515–22. 10.1093/cid/ciy320 29669039 PMC6206106

[25] Ahmed SA, Guerrero Flórez M, Karanis P. The impact of water crises and climate changes on the transmission of protozoan parasites in Africa. Pathogens and global health. 2018;112(6):281–93. 10.1080/20477724.2018.1523778 30332341 PMC6381522

[26] UNICEF. United Nations Children’s Fund. Progress on drinking water and sanitation: 2012 update. 2012. http://www.unicef.org/media/files/JMPreport2012.pdf 2015.

[27] Pullan RL, Freeman MC, Gething PW, Brooker SJ. Geographical inequalities in use of improved drinking water supply and sanitation across sub-Saharan Africa: mapping and spatial analysis of cross-sectional survey data. PLoS Med. 2014;11(4):e1001626 10.1371/journal.pmed.1001626 24714528 PMC3979660

[28] Alyousefi NA, Mahdy MA, Mahmud R, Lim YA. Factors associated with high prevalence of intestinal protozoan infections among patients in Sana’a City, Yemen. PLoS One. 2011;6(7):e22044 10.1371/journal.pone.0022044 21789210 PMC3138770

[29] Anuar TS, Al-Mekhlafi HM, Ghani MKA, Bakar EA, Azreen SN, Salleh FM, et al Molecular epidemiology of amoebiasis in Malaysia: highlighting the different risk factors of Entamoeba histolytica and Entamoeba dispar infections among Orang Asli communities. Int J Parasitol. 2012;42(13–14):1165–75. 10.1016/j.ijpara.2012.10.003 23123168

[30] Bansal D, Gupta P, Singh G, Bhatia M, Singla H. Intestinal Parasitic Infestation in School Going Children of Rishikesh, Uttarakhand, India. Indian Journal of Community Health. 2018;30(1):45–50.

[31] Benetton M, Gonçalves A, Meneghini M, Silva E, Carneiro M. Risk factors for infection by the Entamoeba histolytica/E. dispar complex: an epidemiological study conducted in outpatient clinics in the city of Manaus, Amazon Region, Brazil. Trans R Soc Trop Med Hyg. 2005;99(7):532–40. 10.1016/j.trstmh.2004.11.015 15869773

[32] Blessmann J, Van Linh P, Nu PAT, Thi HD, Muller-Myhsok B, Buss H, et al Epidemiology of amebiasis in a region of high incidence of amebic liver abscess in central Vietnam. The American journal of tropical medicine and hygiene. 2002;66(5):578–83. 10.4269/ajtmh.2002.66.578 12201594

[33] Tuta B-C, Carrero SHS. Prevalencia de parásitos intestinales y factores de riesgo en escolares del colegio Chicamocha Kennedy I del municipio de Tuta, Boyacá-Colombia. 2013.

[34] Dias AP, Calegar D, Carvalho-Costa FA, Alencar MdFL, Ignacio CF, Silva MECd, et al Assessing the Influence of Water Management and Rainfall Seasonality on Water Quality and Intestinal Parasitism in Rural Northeastern Brazil. J Trop Med. 2018;2018 10.1155/2018/8159354 30105057 PMC6076909

[35] Efunshile MA, Ngwu BA, Kurtzhals JA, Sahar S, König B, Stensvold CR. Molecular detection of the carriage rate of four intestinal protozoa with real-time polymerase chain reaction: possible overdiagnosis of Entamoeba histolytica in Nigeria. The American journal of tropical medicine and hygiene. 2015;93(2):257–62. 10.4269/ajtmh.14-0781 26101274 PMC4530744

[36] Fuentes M, Galindez L, Garcia D, Gonzalez N, Goyanes J, Herrera E, et al Frequency of Intestinal Parasitism and Epidemiological Characteristics of the 1 to 12 Year-Old Child Population Treated at the Cerro Gordo Type II Urban Outpatient Clinic. Barquisimeto, State of Lara. January-June 2007. Kasmera. 2011;39(1):31–42.

[37] Hailegebriel T. Prevalence of intestinal parasitic infections and associated risk factors among students at Dona Berber primary school, Bahir Dar, Ethiopia. BMC infectious diseases. 2017;17(1):362 10.1186/s12879-017-2466-x 28535750 PMC5442677

[38] Lander RL, Lander AG, Houghton L, Williams SM, Costa-Ribeiro H, Barreto DL, et al Factors influencing growth and intestinal parasitic infections in preschoolers attending philanthropic daycare centers in Salvador, Northeast Region of Brazil. Cad Saude Publica. 2012;28:2177–88. 10.1590/s0102-311x2012001100017 23147959

[39] Wördemann M, Polman K, Menocal Heredia LT, Junco Diaz R, Collado Madurga AM, Núñez Fernández FA, et al Prevalence and risk factors of intestinal parasites in Cuban children. Trop Med Int Health. 2006;11(12):1813–20. 10.1111/j.1365-3156.2006.01745.x 17176346

[40] Hafedhjameel Z, Al-Amairy AK, Alwatify D. Epidemiologicaland Molecular Study in Patients That Infected with Entamoeba histolytica in Babylon Province. International Journal Of Pharmaceutical Research. 2019;5(2):20–30.

[41] Stroup DF, Berlin JA, Morton SC, Olkin I, Williamson GD, Rennie D, et al Meta-analysis of observational studies in epidemiology: a proposal for reporting. JAMA. 2000;283(15):2008–12. 10.1001/jama.283.15.2008 10789670

[42] Moher D, Liberati A, Tetzlaff J, Altman DG. Preferred reporting items for systematic reviews and meta-analyses: the PRISMA statement. Ann Intern Med. 2009;151(4):264–9. 10.7326/0003-4819-151-4-200908180-00135 19622511

[43] Khaneghah AM, Fakhri Y, Gahruie HH, Niakousari M, Sant’Ana AS. Mycotoxins in cereal-based products during 24 years (1983–2017): a global systematic review. Trends in Food Science & Technology. 2019.

[44] Khaneghah AM, Fakhri Y, Raeisi S, Armoon B, Sant’Ana AS. Prevalence and concentration of ochratoxin A, zearalenone, deoxynivalenol and total aflatoxin in cereal-based products: A systematic review and meta-analysis. Food Chem Toxicol. 2018;118:830–48. 10.1016/j.fct.2018.06.037 29935247

[45] Atamaleki A, Yazdanbakhsh A, Fakhri Y, Mahdipour F, Khodakarim S, Khaneghah AM. The concentration of potentially toxic elements (PTEs) in the onion and tomato irrigated by wastewater: A systematic review; meta-analysis and health risk assessment. Food Research International. 2019:108518 10.1016/j.foodres.2019.108518 31554079

[46] Khaneghah AM, Fakhri Y, Abdi L, Coppa CFSC, Franco LT, de Oliveira CAF. The concentration and prevalence of ochratoxin A in coffee and coffee-based products: a global systematic review, meta-analysis and meta-regression. Fungal Biol. 2019 10.1016/j.funbio.2019.05.012 31345415

[47] Khaneghah AM, Kamani MH, Fakhri Y, Coppa CFSC, de Oliveira CAF, Sant’Ana AS. Changes in masked forms of deoxynivalenol and their co-occurrence with culmorin in cereal-based products: A systematic review and meta-analysis. Food Chem. 2019. 10.1016/j.foodchem.2019.05.034 31126504

[48] Fakhri Y, Gasser R, Rostami A, Fan C, Ghasemi S, Javanian M, et al Toxocara eggs in public places worldwide-A systematic review and meta-analysis. Environmental pollution. 2018;242:1467–75. 10.1016/j.envpol.2018.07.087 30142562

[49] Fakhri Y, Ghorbani R, Taghavi M, Keramati H, Amanidaz N, Moradi B, et al Concentration and Prevalence of Aflatoxin M1 in Human Breast Milk in Iran: Systematic Review, Meta-Analysis, and Carcinogenic Risk Assessment: A Review. J Food Prot. 2019;82(5):785–95. 10.4315/0362-028X.JFP-18-367 30995144

[50] Foroutan M, Fakhri Y, Riahi SM, Ebrahimpour S, Namroodi S, Taghipour A, et al The global seroprevalence of Toxoplasma gondii in pigs: A systematic review and meta-analysis. Vet Parasitol. 2019;269:42–52. 10.1016/j.vetpar.2019.04.012 31079827

[51] Fakhri Y, Rahmani J, Oliveira CAF, Franco LT, Corassin CH, Saba S, et al Aflatoxin M1 in human breast milk: a global systematic review, meta-analysis, and risk assessment study (Monte Carlo simulation). Trends in Food Science & Technology. 2019.

[52] Keusch G, Hamer D, Joe A, Kelley M, Griffiths J, Ward H. Cryptosporidia—who is at risk? Schweizerische Medizinische Wochenschrift. 1995;125(18):899–908. 7770751

[53] Carmena D, Aguinagalde X, Zigorraga C, Fernández‐Crespo J, Ocio J. Presence of Giardia cysts and Cryptosporidium oocysts in drinking water supplies in northern Spain. J Appl Microbiol. 2007;102(3):619–29. 10.1111/j.1365-2672.2006.03193.x 17309610

[54] Morris JA, Gardner MJ. Calculating confidence intervals for relative risks (odds ratios) and standardised ratios and rates. British Medical Journal (Clinical Research Edition). 1988;296(6632):1313–6. 10.1136/bmj.296.6632.1313 PMC2545775 3133061

[55] Strunz EC, Addiss DG, Stocks ME, Ogden S, Utzinger J, Freeman MC. Water, sanitation, hygiene, and soil-transmitted helminth infection: a systematic review and meta-analysis. PLoS Med. 2014;11(3):e1001620 10.1371/journal.pmed.1001620 24667810 PMC3965411

[56] Grade WG. Grading quality of evidence and strength of recommendations. BMJ: British Medical Journal. 2004;328(7454):1490–1. 10.1136/bmj.328.7454.1490 15205295 PMC428525

[57] Atkins D, Eccles M, Flottorp S, Guyatt GH, Henry D, Hill S, et al Systems for grading the quality of evidence and the strength of recommendations I: critical appraisal of existing approaches The GRADE Working Group. BMC Health Serv Res. 2004;4(1):38 10.1186/1472-6963-4-38 15615589 PMC545647

[58] Kuroki T, Watanabe Y, Teranishi H, Izumiyama S, Amemura-Maekawa J, Kura F. Legionella prevalence and risk of legionellosis in Japanese households. Epidemiol Infect. 2017;145(7):1398–408. 10.1017/S0950268817000036 28166862 PMC9203332

[59] Higgins, Thompson S. Quantifying heterogeneity in a meta‐analysis. Stat Med. 2002;21(11):1539–58. 10.1002/sim.1186 12111919

[60] Egger M, Smith GD, Schneider M, Minder C. Bias in meta-analysis detected by a simple, graphical test. BMJ. 1997;315(7109):629–34. 10.1136/bmj.315.7109.629 9310563 PMC2127453

[61] Rahmani J, Alipour S, Miri A, Fakhri Y, Seyed-Mohammad R, Hassan K, et al The prevalence of aflatoxin M1 in milk of Middle East region: A systematic review, meta-analysis and probabilistic health risk assessment. Food Chem Toxicol. 2018;118(2018):653–66. 10.1016/j.fct.2018.06.016 29902498

[62] Graham Clark C. The evolution of Entamoeba, a cautionary tale. Res Microbiol. 2000;151(8):599–603. 10.1016/S0923-2508(00)90127-X. 11081575

[63] Calegar DA, Nunes BC, Monteiro KJL, Santos JPd, Toma HK, Gomes TF, et al Frequency and molecular characterisation of Entamoeba histolytica, Entamoeba dispar, Entamoeba moshkovskii, and Entamoeba hartmanni in the context of water scarcity in northeastern Brazil. Mem Inst Oswaldo Cruz. 2016;111(2):114–9. 10.1590/0074-02760150383 26841049 PMC4750451

[64] Alemu M, Kinfe B, Tadesse D, Mulu W, Hailu T, Yizengaw E. Intestinal parasitosis and anaemia among patients in a Health Center, North Ethiopia. BMC research notes. 2017;10(1):632 10.1186/s13104-017-2957-2 29183355 PMC5704372

[65] Nath J, Ghosh SK, Singha B, Paul J. Molecular epidemiology of amoebiasis: a cross-sectional study among North East Indian population. PLoS neglected tropical diseases. 2015;9(12):e0004225 10.1371/journal.pntd.0004225 26633890 PMC4669114

[66] Dias AP, Calegar D, Carvalho-Costa FA, Alencar MdFL, Ignacio CF, Silva MECd, et al Assessing the Influence of Water Management and Rainfall Seasonality on Water Quality and Intestinal Parasitism in Rural Northeastern Brazil. 2018;2018. 10.1155/2018/8159354 PMC6076909 30105057

[67] Costa JdO Resende JA, Gil FF Santos JFG, Gomes MA. Prevalence of Entamoeba histolytica and other enteral parasitic diseases in the metropolitan region of Belo Horizonte, Brazil. A cross-sectional study. Sao Paulo Med J. 2018;(AHEAD). 10.1590/1516-3180.2018.0036170418 PMC9881705 30110074

[68] Shirley D-A, Hung C-C, Moonah S. Entamoeba histolytica (Amebiasis) Hunter’s Tropical Medicine and Emerging Infectious Diseases: Elsevier; 2020 p. 699–706.

[69] Hooshyar H, Rostamkhani P, Rezaeian M. An Annotated checklist of the human and animal entamoeba (Amoebida: Endamoebidae) species-A review article. Iranian journal of parasitology. 2015;10(2):146 26246811 PMC4522289

[70] Shimokawa C, Kabir M, Taniuchi M, Mondal D, Kobayashi S, Ali IKM, et al Entamoeba moshkovskii is associated with diarrhea in infants and causes diarrhea and colitis in mice. The Journal of infectious diseases. 2012;206(5):744–51. 10.1093/infdis/jis414 22723640 PMC3491739

[71] Singh A, Banerjee T, Kumar R, Shukla SK. Prevalence of cases of amebic liver abscess in a tertiary care centre in India: A study on risk factors, associated microflora and strain variation of Entamoeba histolytica. PloS one. 2019;14(4):e0214880 10.1371/journal.pone.0214880 30943253 PMC6447230

[72] Rollemberg CV, Silva MM, Rollemberg KC, Amorim FR, Lessa NM, Santos MD, et al Predicting frequency distribution and influence of sociodemographic and behavioral risk factors of Schistosoma mansoni infection and analysis of co-infection with intestinal parasites. Geospatial health. 2015 10.4081/gh.2015.303 26054512

[73] Ghssein G, Salami A, Salloum L, Chedid P, Joumaa WH, Fakih H. Surveillance Study of Acute Gastroenteritis Etiologies in Hospitalized Children in South Lebanon (SAGE study). Pediatric gastroenterology, hepatology nutrition 2018;21(3):176–83. 10.5223/pghn.2018.21.3.176 29992117 PMC6037795

[74] Ben Ayed L, Belhassen K, Sabbahi S, Karanis P, Nouiri I, health. Assessment of the parasitological quality of water stored in private cisterns in rural areas of Tunisia. Journal of water health. 2018;16(5):737–49. 10.2166/wh.2018.117 30285955

[75] Mahni MB, Rezaeian M, Eshrat Beigom K, Raeisi A, Khanaliha K, Tarighi F, et al Prevalence of intestinal parasitic infections in Jiroft, Kerman Province, Iran. Iranian journal of parasitology. 2016;11(2):232 28096858 PMC5236101

[76] Alsubaie ASR, Azazy AA, Omer EO, Al-Shibani LA, Al-Mekhlafi AQ, Al-Khawlani FA. Pattern of parasitic infections as public health problem among school children: A comparative study between rural and urban areas. Journal of Taibah University Medical Sciences. 2016;11(1):13–8.

[77] JAMEEL ZH, AL-AMAIRY AK, ALWATIFY D. Epidemiological and Molecular Study in Patients That Infected with Entamoeba histolytica in Babylon Province. International Journal of Pharmaceutical Research. 2019;11(1).

[78] Khan W, Noor un N, Khan A. Prevalence and Risk Factors Associated with Intestinal Parasitic Infections among Food Handlers of Swat, Khyber Pakhtunkhwa, Pakistan. Journal of Food and Nutrition Research. 2019;5(5):331–6.

[79] Berendes D, Kirby A, Clennon JA, Raj S, Yakubu H, Leon J, et al The influence of household-and community-level sanitation and fecal sludge management on urban fecal contamination in households and drains and enteric infection in children. The American journal of tropical medicine hygiene 2017;96(6):1404–14. 10.4269/ajtmh.16-0170 28719269 PMC5462580

[80] Sungkar S, Pohan AP, Ramadani A, Albar N, Azizah F, Nugraha AR, et al Heavy burden of intestinal parasite infections in Kalena Rongo village, a rural area in South West Sumba, eastern part of Indonesia: a cross sectional study. BMC Public Health. 2015;15(1):1296 10.1186/s12889-015-2619-z 26702820 PMC4690433

[81] Nath J, Banyal N, Gautam D, Ghosh S, Singha B, Paul J. Systematic detection and association of Entamoeba species in stool samples from selected sites in India. Epidemiology Infection. 2015;143(1):108–19. 10.1017/S0950268814000715 24703238 PMC9206779

[82] Baker KK, Senesac R, Sewell D, Sen Gupta A, Cumming O, Mumma J. Fecal fingerprints of enteric pathogen contamination in public environments of Kisumu, Kenya, associated with human sanitation conditions and domestic animals. Environmental science technology. 2018;52(18):10263–74. 10.1021/acs.est.8b01528 30106283 PMC6557411

[83] Hemmati N, Razmjou E, Hashemi-Hafshejani S, Motevalian A, Akhlaghi L, Meamar AR. Prevalence and risk factors of human intestinal parasites in Roudehen, Tehran province, Iran. Iranian journal of parasitology. 2017;12(3):364 28979346 PMC5623916

[84] Derso A, Nibret E, Munshea A. Prevalence of intestinal parasitic infections and associated risk factors among pregnant women attending antenatal care center at Felege Hiwot Referral Hospital, northwest Ethiopia. BMC infectious diseases. 2016;16(1):530 10.1186/s12879-016-1859-6 27716099 PMC5045606

[85] Gholipoor Z, Khazan H, Azargashb E, Youssef MR, Rostami A, Health G. Prevalence and risk factors of intestinal parasite infections in Mazandaran province, North of Iran. Clin Epidemiol. 2019.

[86] Khan W, Noor-un-Nisa KA. Prevalence and risk factors associated with intestinal parasitic infections among food handlers of Swat, Khyber Pakhtunkhwa. Pakistan J Food Nutr Res. 2017;5(5):331–6.

[87] Alvarado-Esquivel C, Hernández-Tinoco J, Sánchez-Anguiano LF, Ramos-Nevárez A, Cerrillo-Soto SM, Guido-Arreola CA. Serosurvey of Entamoeba histolytica exposure among tepehuanos population in Durango, Mexico. International journal of biomedical science: IJBS. 2015;11(2):61 26199578 PMC4502734

[88] O’Campo P, Molnar A, Ng E, Renahy E, Mitchell C, Shankardass K, et al Social welfare matters: A realist review of when, how, and why unemployment insurance impacts poverty and health. Soc Sci Med. 2015;132:88–94. 10.1016/j.socscimed.2015.03.025. 25795992

[89] Chin YT, Lim YAL, Chong CW, Teh CSJ, Yap IKS, Lee SC, et al Prevalence and risk factors of intestinal parasitism among two indigenous sub-ethnic groups in Peninsular Malaysia. Infectious diseases of poverty. 2016;5(1):77 10.1186/s40249-016-0168-z 27430215 PMC4950084

[90] Organization WH. Research priorities for zoonoses and marginalized infections: World Health Organization; 2012. 23420951

[91] Liao C-W, Chiu K-C, Chiang I-C, Cheng P-C, Chuang T-W, Kuo J-H, et al Prevalence and risk factors for intestinal parasitic infection in schoolchildren in Battambang, Cambodia. The American journal of tropical medicine. 2017;96(3):583–8. 10.4269/ajtmh.16-0681 28070012 PMC5361531

[92] Lochs H, Allison SP, Meier R, Pirlich M, Kondrup J, Schneider S, et al Introductory to the ESPEN Guidelines on Enteral Nutrition: Terminology, Definitions and General Topics. Clin Nutr. 2006;25(2):180–6. 10.1016/j.clnu.2006.02.007. 16697086

[93] Raja C, Karthick P. Role of alcoholism in liver abscess. Int J Res Med Sci. 2014;2:1313–9.

[94] Kannathasan S, Murugananthan A, Kumanan T, de Silva NR, Rajeshkannan N, Haque R, et al Epidemiology and factors associated with amoebic liver abscess in northern Sri Lanka. BMC public health. 2018;18(1):118 10.1186/s12889-018-5036-2 29316900 PMC5761098

[95] Casavechia MTG, Lonardoni MVC, Venazzi EAS, Campanerut-Sá PAZ, da Costa Benalia HR, Mattiello MF, et al Prevalence and predictors associated with intestinal infections by protozoa and helminths in southern Brazil. Parasitology research. 2016;115(6):2321–9. 10.1007/s00436-016-4980-y 26987643

[96] Fonseca REPd Barbosa MCR, Ferreira BR. High prevalence of enteroparasites in children from Ribeirão Preto, São Paulo, Brazil. Revista brasileira de enfermagem. 2017;70(3):566–71. 10.1590/0034-7167-2016-0059 28562805

[97] Suntaravitun P, Dokmaikaw A. Prevalence of Intestinal Parasites and Associated Risk Factors for Infection among Rural Communities of Chachoengsao Province, Thailand. The Korean journal of parasitology. 2018;56(1):33 10.3347/kjp.2018.56.1.33 29529848 PMC5858660

[98] Shahdoust S, Niyyati M, Haghighi A, Azargashb E, Khataminejad MR. Prevalence of intestinal parasites in referred individuals to the medical centers of Tonekabon city, Mazandaran province. Gastroenterology hepatology from bed to bench. 2016;9(Suppl1):S75 28224032 PMC5310804

[99] Chaves RD, Pradella F, Turatti MA, Amaro EC, da Silva AR, dos Santos Farias A, et al Evaluation of Staphylococcus spp. in food and kitchen premises of Campinas, Brazil. Food control. 2018;84:463–70.

[100] Coffey D, Gupta A, Hathi P, Spears D, Srivastav N, Vyas S. Culture and the health transition: understanding sanitation behavior in rural north India. International Growth Centre Working Paper, April. 2015.

[101] Petney TN. Environmental, cultural and social changes and their influence on parasite infections. Int J Parasitol. 2001;31(9):919–32. 10.1016/S0020-7519(01)00196-5. 11406141

[102] Okeke IN, Lamikanra A, Edelman R. Socioeconomic and behavioral factors leading to acquired bacterial resistance to antibiotics in developing countries. Emerg Infect Dis. 1999;5(1):18 10.3201/eid0501.990103 10081668 PMC2627681

[103] Alanazi A. The prevalence of intestinal parasitic protozoan among patients in Ad-Dawadimi General Hospital, Saudi Arabia. Tropical Biomedicine. 2017;34(2):453–60. 33593028

[104] Cissé G. Food-borne and water-borne diseases under climate change in low- and middle-income countries: Further efforts needed for reducing environmental health exposure risks. Acta Tropica. 2019;194:181–8. 10.1016/j.actatropica.2019.03.012. 30946811 PMC7172250

[105] Morley NJ, Lewis JW. Temperature stress and parasitism of endothermic hosts under climate change. Trends in Parasitology. 2014;30(5):221–7. 10.1016/j.pt.2014.01.007. 24613288

[106] Tengku S, Norhayati M. Review Paper Public health and clinical importance of amoebiasis in Malaysia: a review. Trop Biomed. 2011;28:194–222. 22041740

[107] Njuguna C, Njeru I, Mgamb E, Langat D, Makokha A, Ongore D, et al Enteric pathogens and factors associated with acute bloody diarrhoea, Kenya. J BMC infectious diseases. 2016;16(1):477. 10.1186/s12879-016-1814-6 PMC5012060 27600526

[108] UNICEF D. Monitoring the situation of children and women. 2005.

[109] Al-Ansari N. Management of water resources in Iraq: perspectives and prognoses. Engineering. 2013;5(6):667–84.

[110] Fewtrell L, Bartram J. Water quality: guidelines, standards & health: IWA publishing; 2001.

[111] de la Luz Galván-Ramírez M, Madriz-Elisondo AL, Ramírez CGT, Rameño JdJR, de la O Carrasco DA, López MAC. Enteroparasitism and Risk Factors Associated with Clinical Manifestations in Children and Adults of Jalisco State in Western Mexico. Osong public health research perspectives. 2019;10(1):39 10.24171/j.phrp.2019.10.1.08 30847270 PMC6396823

[112] Quihui L, Valencia ME, Crompton DW, Phillips S, Hagan P, Morales G, et al Role of the employment status and education of mothers in the prevalence of intestinal parasitic infections in Mexican rural schoolchildren. BMC Public Health. 2006;6(1):225 10.1186/1471-2458-6-225 16956417 PMC1584408

