# Supplementary material for: The association between the lack of safe drinking water and sanitation facilities with intestinal Entamoeba spp infection risk: A systematic review and meta-analysis
Source: PLoS One. 2020 Nov 4;15(11):e0237102. doi: 10.1371/journal.pone.0237102 (PMC7641376; doi:10.1371/journal.pone.0237102)
Supplement: S1 Table — (DOCX) [file pone.0237102.s002.docx]

S1 Table. Main characteristic of the included studies

|  |  |  |  |  |  |  |  |  |  | Information about rating | | | |  |
| --- | --- | --- | --- | --- | --- | --- | --- | --- | --- | --- | --- | --- | --- | --- |
| Study design | **Toilet/water treatment** | **Odds** | **Lower** | **Upper** | **Country** | **Study year** | **Age group** | **Intestinal protozoa species** | **Data obtained** | **Diagnostic** | **Toilet status or water treatment assessment method** | **Other strengths and limitations** | **Total points** | **Reference** |
| C/S^[[1]](#footnote-1)^ | Safe drinking water lack | 1.66 | 0.54 | 2.69 | Yemen | NM | All | EH/ED | OR | 2 | 0 | 0 | 2 | [[1](#_ENREF_1)] |
| C/S | Sanitation (toilet) facilities lack | 7.47 | 1.82 | 30.65 | India | NM | Children | EH | 2*2 t | 1 | 0 | 0 | 1 | [[2](#_ENREF_2)] |
| C/S | Safe drinking water lack | 0.53 | 0.42 | 5.68 | India | NM | Children | EH | 2*2 t | 1 | 0 | 0 | 1 | [[2](#_ENREF_2)] |
| C/C^[[2]](#footnote-2)^ | Sanitation (toilet) facilities lack | 1.01 | 0.7 | 1.44 | Brazil | 2001-2002 | All | EH/ED | 2*2 t | 3 | 0 | 0 | 3 | [[3](#_ENREF_3)] |
| C/C | Safe drinking water lack | 1.32 | 1.01 | 1.73 | Brazil | 2001-2002 | All | EH/ED | 2*2 t | 3 | 0 | 0 | 3 | [[3](#_ENREF_3)] |
| C/S | Sanitation (toilet) facilities lack | 2.44 | 1.36 | 4.37 | Vietnam | 1999 | Adult | EH | 2*2 t | 2 | 0 | 0 | 2 | [[4](#_ENREF_4)] |
| C/S | Sanitation (toilet) facilities lack | 2.44 | 0.98 | 6.07 | Brazil | 2013 | All | EH/ED/EM | 2*2 t | 1 | 0 | 0 | 2 | [[5](#_ENREF_5)] |
| C/S | Safe drinking water lack | 0.64 | 0.11 | 3.42 | Colombia | NM | Children | EH/ED | 2*2 t | 1 | 0 | 0 | 1 | [[6](#_ENREF_6)] |
| C/S | Sanitation (toilet) facilities lack | 0.63 | 0.07 | 5.26 | Brazil | 2014 | All | EH/ED | 2*2 t | 2 | 0 | 1 | 3 | **[**[**7**](#_ENREF_7)**]** |
| C/S | Sanitation (toilet) facilities lack | 2.28 | 0.68 | 7.89 | Brazil | 2015 | All | EH/ED | 2*2 t | 2 | 0 | 1 | 3 | **[**[**7**](#_ENREF_7)**]** |
| C/S | Sanitation (toilet) facilities lack | 1.66 | 0.8 | 3.48 | Brazil | 2016 | All | EH/ED | 2*2 t | 2 | 0 | 1 | 3 | **[**[**7**](#_ENREF_7)**]** |
| C/S | Sanitation (toilet) facilities lack | 1.48 | 0.48 | 4.49 | Nigeria | NM | Children | ED | 2*2 t | 2 | 0 | 0 | 2 | [[8](#_ENREF_8)] |
| C/S | Sanitation (toilet) facilities lack | 2.02 | 0.38 | 10.57 | Lesotho | 2007 | Children | EH/ED | 2*2 t | 1 | 0 | 0 | 1 | [[9](#_ENREF_9)] |
| C/S | Safe drinking water lack | 0.14 | 0.01 | 1.66 | Lesotho | 2007 | Children | EH/ED | 2*2 t | 1 | 0 | 0 | 1 | [[9](#_ENREF_9)] |
| C/S | Sanitation (toilet) facilities lack | 0.88 | 0.45 | 1.74 | Ethiopia | 2015-2016 | Children | EH | OR | 2 | 0 | 0 | 2 | **[**[**10**](#_ENREF_10)**]** |
| C/S | Safe drinking water lack | 0.78 | 0.44 | 1.41 | Cambodia | 2015 | Children | EH | 2*2 t | 1 | 0 | 0 | 1 | [[11](#_ENREF_11)] |
| C/S | Sanitation (toilet) facilities lack | 1.89 | 1.21 | 3.57 | Ethiopia | NM | Adult | EH/ED | OR | 2 | 0 | 1 | 3 | [[12](#_ENREF_12)] |
| C/S | Sanitation (toilet) facilities lack | 1.21 | 1.01 | 1.45 | India | NM | Adult | EH/ED | OR | 2 | 0 | 1 | 3 | [[13](#_ENREF_13)] |
| C/S | Sanitation (toilet) facilities lack | 0.96 | 0.54 | 1.71 | Cote dlvoire | 2004-2005 | All | EH/ED | OR | 2 | 0 | 1 | 3 | [[14](#_ENREF_14)] |
| C/S | Sanitation (toilet) facilities lack | 1.18 | 0.65 | 2.18 | Uganda | 2011 | All | EH/ED | 2*2 t | 1 | 0 | 0 | 1 | [[15](#_ENREF_15)] |
| C/S | Sanitation (toilet) facilities lack | 1.13 | 0.63 | 2.05 | Mexico | 1998 | Children | EH/ED | OR | 2 | 0 | 0 | 2 | [[16](#_ENREF_16)] |
| C/S | Sanitation (toilet) facilities lack | 1.98 | 1.18 | 3.33 | India | NM | All | EH/ED | OR | 2 | 0 | 1 | 3 | **[**[**17**](#_ENREF_17)**]** |
| C/S | Safe drinking water lack | 2.8 | 1.53 | 5.1 | India | NM | All | EH/ED | OR | 2 | 0 | 1 | 3 | **[**[**17**](#_ENREF_17)**]** |
| C/S | Sanitation (toilet) facilities lack | 1.67 | 0.33 | 8.32 | South Africa | 2009 | Children | EH/ED | 2*2 t | 2 | 0 | 0 | 2 | [[18](#_ENREF_18)] |
| C/S | Sanitation (toilet) facilities lack | 1.31 | 0.25 | 6.75 | Vietnam | 2008 | All | EH/ED | 2*2 t | 2 | 0 | 0 | 2 | [[19](#_ENREF_19)] |
| C/S | Sanitation (toilet) facilities lack | 18.2 | 7.49 | 22.2 | Mexico | 1997-1998 | Children | EH/ED | 2*2 t | 2 | 0 | 0 | 2 | [[20](#_ENREF_20)] |
| C/S | Sanitation (toilet) facilities lack | 1.92 | 1.01 | 3.58 | India | 1970 | All | EH/ED | 2*2 t | 2 | 1 | -1 | 2 | [[21](#_ENREF_21)] |
| C/S | Sanitation (toilet) facilities lack | 0.98 | 0.58 | 1.66 | Ecuador | 2003 | Children | EH/ED | 2*2 t | 1 | 0 | 0 | 1 | [[22](#_ENREF_22)] |
| C/S | Sanitation (toilet) facilities lack | 1.09 | 0.78 | 1.52 | Cote dlvoire | 2011 | All | EH/ED | 2*2 t | 2 | 0 | 1 | 3 | [[23](#_ENREF_23)] |
| C/S | Safe drinking water lack | 6.4 | 1.9 | 21.2 | India | 2017 | All | EH | OR | 2 | 0 | 1 | 3 | **[**[**24**](#_ENREF_24)**]** |
| C/S | Sanitation (toilet) facilities lack | 1.53 | 0.63 | 3.69 | Ethiopia | 2007 | All | EH/ED | 2*2 t | 2 | 0 | 0 | 2 | [[25](#_ENREF_25)] |
| C/S | Sanitation (toilet) facilities lack | 1.58 | 0.75 | 3.35 | Chile | 1987 | All | EH/ED | 2*2 t | 1 | 0 | 0 | 1 | [[26](#_ENREF_26)] |
| C/S | Sanitation (toilet) facilities lack | 12.9 | 4.04 | 25.05 | Kenya | 2009-2010 | Adult | EH/ED | 2*2 t | 2 | 0 | 0 | 2 | [[27](#_ENREF_27)] |
| C/S | Safe drinking water lack | 2.76 | 0.62 | 12.73 | Kenya | 2009-2010 | Adult | EH/ED | 2*2 t | 2 | 0 | 0 | 2 | [[27](#_ENREF_27)] |
| C/S | Safe drinking water lack | 0.51 | 0.45 | 1.35 | Cuba | 2003-2004 | Children | EH/ED | 2*2 t | 2 | 0 | 0 | 2 | [[28](#_ENREF_28)] |
| C/S | Safe drinking water lack | 2.04 | 0.95 | 4.37 | Iraq | 2017-2018 | All | EH | 2*2 t | 1 | 0 | 0 | 1 | **[**[**29**](#_ENREF_29)**]** |

**References**

1. Alyousefi NA, Mahdy MA, Mahmud R, Lim YA. Factors associated with high prevalence of intestinal protozoan infections among patients in Sana'a City, Yemen. PLoS One. 2011;6(7):e22044.

2. Bansal D, Gupta P, Singh G, Bhatia M, Singla H. Intestinal Parasitic Infestation in School Going Children of Rishikesh, Uttarakhand, India. Indian Journal of Community Health. 2018;30(1):45-50.

3. Benetton M, Gonçalves A, Meneghini M, Silva E, Carneiro M. Risk factors for infection by the Entamoeba histolytica/E. dispar complex: an epidemiological study conducted in outpatient clinics in the city of Manaus, Amazon Region, Brazil. Trans R Soc Trop Med Hyg. 2005;99(7):532-40.

4. Blessmann J, Van Linh P, Nu PAT, Thi HD, Muller-Myhsok B, Buss H, et al. Epidemiology of amebiasis in a region of high incidence of amebic liver abscess in central Vietnam. The American journal of tropical medicine and hygiene. 2002;66(5):578-83.

5. Calegar DA, Nunes BC, Monteiro KJL, Santos JPd, Toma HK, Gomes TF, et al. Frequency and molecular characterisation of Entamoeba histolytica, Entamoeba dispar, Entamoeba moshkovskii, and Entamoeba hartmanni in the context of water scarcity in northeastern Brazil. Mem Inst Oswaldo Cruz. 2016;111(2):114-9.

6. Tuta B-C, Carrero SHS. Prevalencia de parásitos intestinales y factores de riesgo en escolares del colegio Chicamocha Kennedy I del municipio de Tuta, Boyacá-Colombia. 2013.

7. Dias AP, Calegar D, Carvalho-Costa FA, Alencar MdFL, Ignacio CF, Silva MECd, et al. Assessing the Influence of Water Management and Rainfall Seasonality on Water Quality and Intestinal Parasitism in Rural Northeastern Brazil. J Trop Med. 2018;2018.

8. Efunshile MA, Ngwu BA, Kurtzhals JA, Sahar S, König B, Stensvold CR. Molecular detection of the carriage rate of four intestinal protozoa with real-time polymerase chain reaction: possible overdiagnosis of Entamoeba histolytica in Nigeria. The American journal of tropical medicine and hygiene. 2015;93(2):257-62.

9. Fuentes M, Galindez L, Garcia D, Gonzalez N, Goyanes J, Herrera E, et al. Frequency of Intestinal Parasitism and Epidemiological Characteristics of the 1 to 12 Year-Old Child Population Treated at the Cerro Gordo Type II Urban Outpatient Clinic. Barquisimeto, State of Lara. January-June 2007. Kasmera. 2011;39(1):31-42.

10. Hailegebriel T. Prevalence of intestinal parasitic infections and associated risk factors among students at Dona Berber primary school, Bahir Dar, Ethiopia. BMC Infect Dis. 2017;17(1):362.

11. Liao C-W, Chiu K-C, Chiang I-C, Cheng P-C, Chuang T-W, Kuo J-H, et al. Prevalence and risk factors for intestinal parasitic infection in schoolchildren in Battambang, Cambodia. The American journal of tropical medicine and hygiene. 2017;96(3):583-8.

12. Mahmud MA, Bezabih AM, Gebru RB. Risk factors for intestinal parasitosis among antiretroviral-treated HIV/AIDS patients in Ethiopia. Int J STD AIDS. 2014;25(11):778-84.

13. Mathur T, Kaur J. The epidemiology of amoebiasis in an urban area. Indian J Med Res. 1972;60(8):1134-7.

14. Matthys B, Tschannen AB, Tian‐Bi NT, Comoé H, Diabaté S, Traoré M, et al. Risk factors for Schistosoma mansoni and hookworm in urban farming communities in western Côte d'Ivoire. Trop Med Int Health. 2007;12(6):709-23.

15. McElligott JT, Naaktgeboren C, Makuma-Massa H, Summer AP, Deal JL. Prevalence of intestinal protozoa in communities along the Lake Victoria region of Uganda. Int J Infect Dis. 2013;17(8):e658-e9.

16. Morales EM, Sánchez HJ, del Mar García M, Vargas G, Méndez JD, Pérez M. Intestinal parasites in children, in highly deprived areas in the border region of Chiapas, Mexico. Salud Publica Mex. 2003;45(5):379-88.

17. Nath J, Banyal N, Gautam D, Ghosh S, Singha B, Paul J. Systematic detection and association of Entamoeba species in stool samples from selected sites in India. Epidemiol Infect. 2015;143(1):108-19.

18. Nxasana N, Baba K, Bhat V, Vasaikar S. Prevalence of intestinal parasites in primary school children of Mthatha, Eastern Cape Province, South Africa. Annals of medical and health sciences research. 2013;3(4):511-6.

19. Duc PP, Nguyen-Viet H, Hattendorf J, Zinsstag J, Cam PD, Odermatt P. Risk factors for Entamoeba histolytica infection in an agricultural community in Hanam province, Vietnam. Parasites & vectors. 2011;4(1):102.

20. Quihui L, Valencia ME, Crompton DW, Phillips S, Hagan P, Morales G, et al. Role of the employment status and education of mothers in the prevalence of intestinal parasitic infections in Mexican rural schoolchildren. BMC Public Health. 2006;6(1):225.

21. Rao C, Krishnaswami A, Gupta S, Biswas H, Raghavan N. Prevalence of amoebiasis and other intestinal parasitic infections in a selected community. Indian J Med Res. 1971;59(9).

22. Rinne S, Rodas EJ, Galer-Unti R, Glickman N, Glickman LT. Prevalence and risk factors for protozoan and nematode infections among children in an Ecuadorian highland community. Trans R Soc Trop Med Hyg. 2005;99(8):585-92.

23. Schmidlin T, Hürlimann E, Silué KD, Yapi RB, Houngbedji C, Kouadio BA, et al. Effects of hygiene and defecation behavior on helminths and intestinal protozoa infections in Taabo, Côte d’Ivoire. PLoS One. 2013;8(6):e65722.

24. Singh A, Banerjee T, Kumar R, Shukla SK. Prevalence of cases of amebic liver abscess in a tertiary care centre in India: A study on risk factors, associated microflora and strain variation of Entamoeba histolytica. PLoS One. 2019;14(4):e0214880.

25. Taye B, Desta K, Ejigu S, Dori GU. The magnitude and risk factors of intestinal parasitic infection in relation to Human Immunodeficiency Virus infection and immune status, at ALERT Hospital, Addis Ababa, Ethiopia. Parasitol Int. 2014;63(3):550-6.

26. Torres P, Miranda JC, Flores L, Riquelme J, Franjola R, Perez J, et al. Blastocystosis and other intestinal protozoan infections in human riverside communities from-Valdivia River Basin, Chile. Rev Inst Med Trop Sao Paulo. 1992;34(6):557-64.

27. Wanyiri JW, Kanyi H, Maina S, Wang DE, Ngugi P, O'Connor R, et al. Infectious diarrhoea in antiretroviral therapy-naive HIV/AIDS patients in Kenya. Trans R Soc Trop Med Hyg. 2013;107(10):631-8.

28. Wördemann M, Polman K, Menocal Heredia LT, Junco Diaz R, Collado Madurga AM, Núñez Fernández FA, et al. Prevalence and risk factors of intestinal parasites in Cuban children. Trop Med Int Health. 2006;11(12):1813-20.

29. Hafedhjameel Z, Al-Amairy AK, Alwatify D. Epidemiologicaland Molecular Study in Patients That Infected with Entamoeba histolytica in Babylon Province. International Journal Of Pharmaceutical Research. 2019;5(2):20-30.

1. Cross-sectional [↑](#footnote-ref-1)
2. Case-control [↑](#footnote-ref-2)
