# Supplementary material for: The association between the lack of safe drinking water and sanitation facilities with intestinal Entamoeba spp infection risk: A systematic review and meta-analysis
Source: PLoS One. 2020 Nov 4;15(11):e0237102. doi: 10.1371/journal.pone.0237102 (PMC7641376; doi:10.1371/journal.pone.0237102)
Supplement: S1 Text — (DOCX) [file pone.0237102.s003.docx]

| Limitations | Limitations current study were including (1) Despite a comprehensive review in many countries of the world no study was found and (2) In addition, in some countries, one study was conducted that made it difficult to interpret the data. |
| --- | --- |
| Search strategy in Scopus | ( ( TITLE-ABS-KEY ( water )  OR  TITLE-ABS-KEY ( "toilet facilities" )  OR  TITLE-ABS-KEY ( sanitation ) )  AND  DOCTYPE ( ar ) )  AND  ( ( TITLE-ABS-KEY ( "relative risk" )  OR  TITLE-ABS-KEY ( risk )  OR  TITLE-ABS-KEY ( "odds ratio" )  OR  TITLE-ABS-KEY ( "risk factor" ) )  AND  DOCTYPE ( ar ) )  AND  ( ( TITLE-ABS-KEY ( "Intestinal Protozoa" )  OR  TITLE-ABS-KEY ( "Entamoeba histolytica‏" )  OR  TITLE-ABS-KEY ( "Entamoeba dispar" )  OR  TITLE-ABS-KEY ( "Entamoeba moshkovskii" ) )  AND  DOCTYPE ( ar ) ) |
| Bias | Publication bias that detected via Egger's test |
