# Supplementary material for: The association between the lack of safe drinking water and sanitation facilities with intestinal Entamoeba spp infection risk: A systematic review and meta-analysis
Source: PLoS One. 2020 Nov 4;15(11):e0237102. doi: 10.1371/journal.pone.0237102 (PMC7641376; doi:10.1371/journal.pone.0237102)
Supplement: S4 Fig — ES is Effect size. (DOCX) [file pone.0237102.s007.docx]

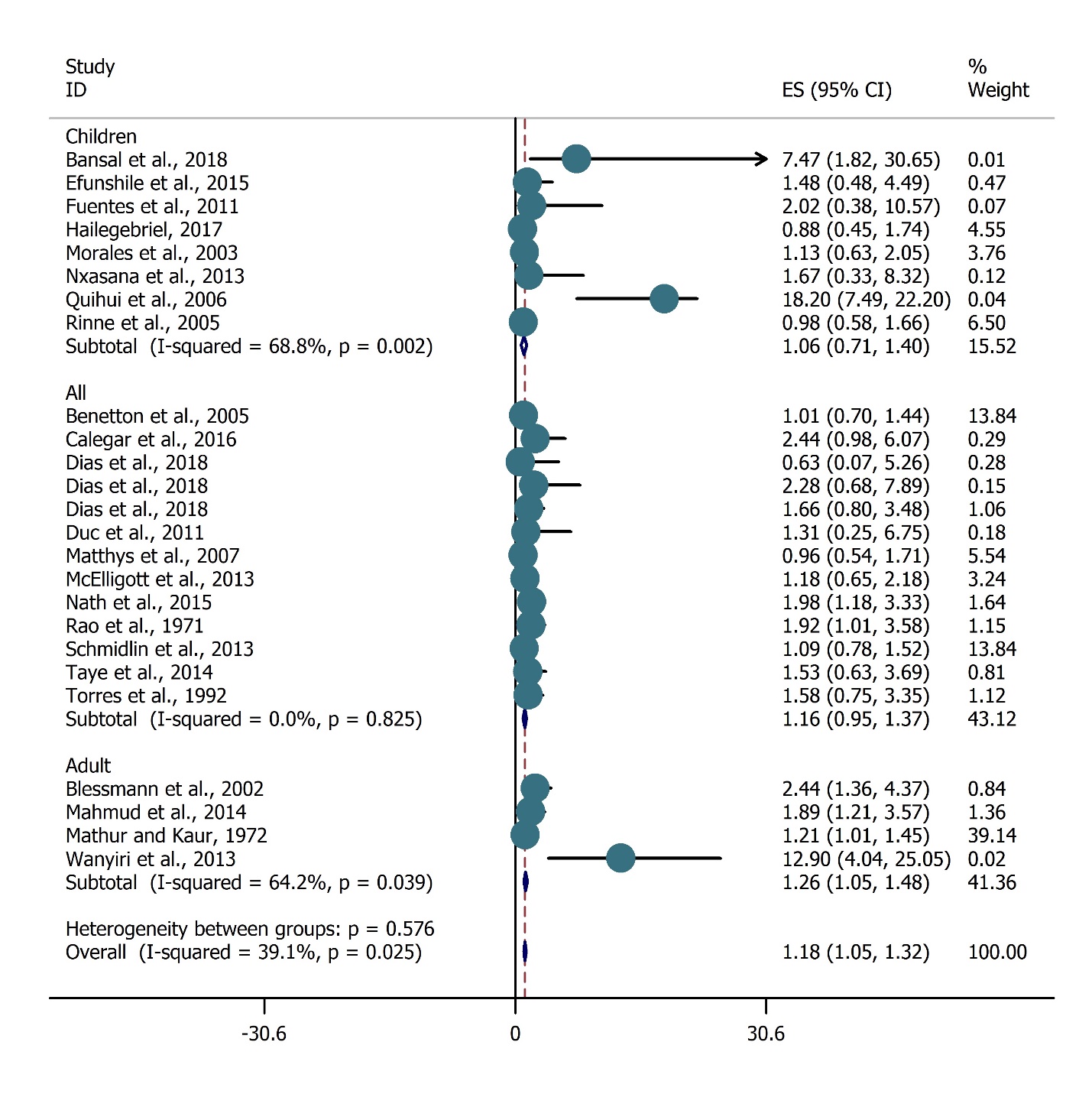


**S4 Fig.** Meta-analysis of the association intestinal *Entamoeba spp* infection with lack sanitation facilities (toilet) based on age group subgroup. ES is Effect size.
